# Targeting Pivotal Hallmarks of Cancer for Enhanced Therapeutic Strategies in Triple-Negative Breast Cancer Treatment—In Vitro, In Vivo and Clinical Trials Literature Review

**DOI:** 10.3390/cancers16081483

**Published:** 2024-04-12

**Authors:** Anna Szulc, Marta Woźniak

**Affiliations:** Department of Clinical and Experimental Pathology, Division of General and Experimental Pathology, Wroclaw Medical University, 50-368 Wroclaw, Poland; anna.szulc@umw.edu.pl

**Keywords:** Warburg effect, triple-negative breast cancer, targeted therapy, glycoconjugates

## Abstract

**Simple Summary:**

This literature review explores the potential of targeted therapies in triple-negative breast cancer (TNBC) treatment, focusing on distinct features of cancer cells. This article delves into the latest advancements in therapeutic strategies targeting components of the tumor microenvironment and pivotal hallmarks of cancer: deregulating cellular metabolism and the Warburg effect, acidosis and hypoxia, the ability to metastasize and evade the immune system, aiming to enhance treatment efficacy while mitigating systemic toxicity. Our study aims to provide the most up-to-date review of in vitro and in vivo studies and clinical trials, highlighting the promising effectiveness, elucidating the mechanisms, and identifying the limitations of novel targeted therapies. Results reveal that while many therapies are in the preclinical phase, requiring further investigation, CAR-T therapy has progressed to clinical trials. However, there remains a lack of data on patient response to this therapy. Nonetheless, the integration of targeted therapies tailored to TNBC’s molecular characteristics holds significant potential for optimizing clinical outcomes. These conclusions underscore the importance of ongoing research in advancing treatment options for TNBC, addressing the critical need for more effective therapies in clinical practice, and ultimately improving patient outcomes and quality of life.

**Abstract:**

This literature review provides a comprehensive overview of triple-negative breast cancer (TNBC) and explores innovative targeted therapies focused on specific hallmarks of cancer cells, aiming to revolutionize breast cancer treatment. TNBC, characterized by its lack of expression of estrogen receptor (ER), progesterone receptor (PR), and human epidermal growth factor receptor 2 (HER2), presents distinct features, categorizing these invasive breast tumors into various phenotypes delineated by key elements in molecular assays. This article delves into the latest advancements in therapeutic strategies targeting components of the tumor microenvironment and pivotal hallmarks of cancer: deregulating cellular metabolism and the Warburg effect, acidosis and hypoxia, the ability to metastasize and evade the immune system, aiming to enhance treatment efficacy while mitigating systemic toxicity. Insights from in vitro and in vivo studies and clinical trials underscore the promising effectiveness and elucidate the mechanisms of action of these novel therapeutic interventions for TNBC, particularly in cases refractory to conventional treatments. The integration of targeted therapies tailored to the molecular characteristics of TNBC holds significant potential for optimizing clinical outcomes and addressing the pressing need for more effective treatment options for this aggressive subtype of breast cancer.

## 1. Introduction

### 1.1. Hallmarks of Cancer

Cancer types are characterized by remarkable diversity; nevertheless, they are united by shared commonalities, which have been labeled as the hallmarks of cancer. These are traits that human cells acquire during their progression from normal to malignant form, enabling them to evade the tissue-based restrictions on their growth by sustaining the primary pro-survival and pro-proliferative signals consistently in the active mode. Thus, they attain access to limitless proliferation, resulting in tumor formation and specific microenvironment creation.

The cornerstone of this conception was first depicted in “The Hallmarks of Cancer” by Douglas Hanahan and Robert Weinberg, published in January 2000 in “Cell”, who announced six biological capabilities crucial for neoplastic development [1]. They comprised sustaining proliferative signaling, evading growth suppressors, resisting cell death, enabling replicative immortality, inducing angiogenesis, and activating invasion and metastasis [2]. Later, in 2011, they were expanded to eight when two other core hallmarks emerged: deregulating cellular metabolism and avoiding immune destruction [3]. In March 2023, an updated list appeared in “Hallmarks of Cancer Handbook”, adding unlocking phenotypic plasticity, epigenetic reprograming, tumor-promoting inflammation, polymorphic microbiomes, senescent cells, genome instability, and mutation to the vast complexity of phenotypes and genotypes.

### 1.2. Tripple-Negative Breast Cancer (TNBC)

It is well known that irregularities, along with inheritable genetic alterations in germinal or somatic cells, result in malignancy. Similarly, a combination of environmental and genetic factors leads to the accumulation of mutations in particular genes, triggering breast cancer (BC) [4]. According to the European Cancer Inequalities Registry in Poland, 366 new cases of breast cancer were diagnosed in 2020, making it the most common cancer among women [5]. With a total of 2.26 million cases reported in 2020, breast cancer is the most frequently diagnosed disease worldwide and the primary cause of cancer-related deaths among women. As such, it poses a serious threat to global [6].

A novel gene expression-based classification system for breast cancer divided breast tumors into six “intrinsic subtypes”: luminal subtype (subdivided into Luminal A and Luminal B), human epidermal growth factor receptor-2 (HER2) subtype, basal-like, normal breast-like, and Claudin-low [7,8]. Approximately 10–20% of invasive breast tumors are classified as TNBC, which encompasses several molecular subtypes. The phenotype referred to as triple negative has key elements in molecular assays, including normal breast-like cancers, basal-like malignancies, and the more recently identified subtypes with low claudin levels. Breast cancers with BRCA1/2 gene mutations are also included [9]. All in all, the enumerated types are characterized by the absence of estrogen receptor (ER) and progesterone receptor (PR) and the lack of HER2 overexpression or amplification [10]. Moreover, TNBC is prevalent in premenopausal women, proliferates more rapidly, and exhibits high aggressiveness in comparison to hormone receptor-positive (HR+) cancers. It is linked to poor overall survival, a high frequency of distant metastases, and high recurrence rates [11,12]. 

The lack of the estrogen receptor (ER), progesterone receptor (PR), and HER2 receptor renders patients with this malignancy not eligible for hormone or anti-HER2 therapy; hence, chemotherapy remains the standard of care [13]. Admittedly, in the early stages of TNBC, chemotherapy appears to be the best choice for the action on fast-growing cells, notwithstanding that it leads to unsatisfactory long-term results due to systemic toxicity [14]. Chemotherapy is one of the components of complex treatment, along with surgical excision of the tumor, including any regional metastases, and radiation therapy applied topically [15]. Surgical options vary from a radical mastectomy (RM) to organ-saving operations. The former frequently necessitates the removal of a large section of the breast, which results in significant body deformations and unsatisfactory aesthetic outcomes, taking its toll on the patient’s physical appearance and mental health [16,17]. In recent years, there has been a general tendency towards a combination of oncology and plastic surgery, with an increase in the number of cases of skin-preserving mastectomy using plastic prostheses or own tissues, as well as a shift away from massive lymph node dissections and towards sentinel lymph node biopsies [18]. 

Having remarked on adjuvant and postoperative systemic therapy, we would like to discuss neoadjuvant therapy, which refers to the use of systemic treatment prior to surgery. Neoadjuvant therapy was first employed when treating locally advanced, incurable breast cancer. Consequently, a great deal of research has been performed on the efficacy of neoadjuvant therapy in patients with operable breast cancer. There was interest in applying preoperative systemic therapy to reduce the extent and morbidity of curative surgery, building on the observation that systemic treatment could make certain inoperable patients eligible for surgery [19]. Due to the initial chemosensitivity of TNBC, several strategies have been pursued to increase the pathological complete response (pCR) of neoadjuvant chemotherapy [20]. 

Due to numerous factors, including increased immunogenicity, higher enrichment in tumor-infiltrating lymphocytes (TILs), and a greater quantity of programed cell death ligand 1 (PD-L1) expression, TNBC is more likely than other breast cancer subtypes to benefit from immune checkpoint blockade therapy [21]. In 2019, the US Food and Drug Administration (FDA) approved atezolizumab, an anti-PD-L1 antibody, in combination with nanoparticle albumin-bound (nab)-paclitaxel as a first-line therapy targeting the factor sustaining immunosuppression in the tumor environment [22]. As of 2023, the FDA has approved neoadjuvant pembrolizumab, likewise an anti-PD-1 drug, with chemotherapy for patients with early TNBC, based on randomized phase III trial testing, which demonstrated that the pCR rate was significantly increased and, more importantly, the risk of recurrence was reduced for all patients, regardless of PD-L1 expression [23]. 

As for potential therapeutics currently undergoing clinical trials, poly-ADP ribose polymerase (PARP) inhibitors, such as olaparib, veliparib, and rucaparib, signal promising outcomes for basal-like 1 subtype patients. The nuclear enzyme PARP is primarily involved in DNA repair, genome stabilization, cell cycle progression, apoptosis, and transcription regulation. PARP inhibitors decrease DNA repair activity and promote apoptosis [24]. They enhance tumor synthetic lethality in people with BRCA1/2 gene mutations and significantly increase the advantages of chemotherapy treatment. Treatment for BRCA1/2-deficient cancers frequently involves the use of PARP inhibitors, which exhibit substantial anticancer effects in this context that considerably outweigh those for BRCA1-expressing tumors [25]. 

### 1.3. Aim of This Study

**Figure 1:**
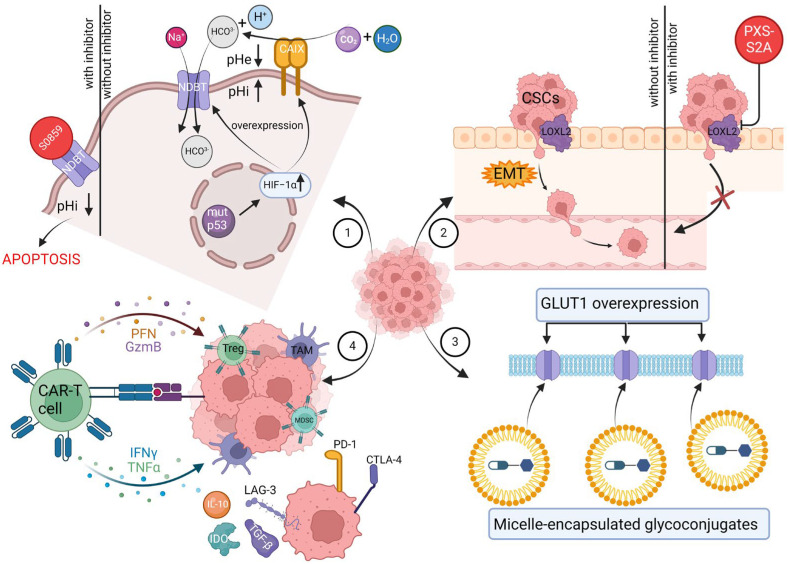
Four main pathways for breast cancer treatment. 1. Inhibiting sodium-driven bicarbonate transporters (NDBTs) decreases intracellular pH (pHi) leading to tumor cell apoptosis; pHe—extracellular pH, pHi—intracellular pH. 2. Inhibiting lysyl oxidase homolog 2 (LOXL2) suppresses cellular proliferation. 3. Micelle-encapsulated glycoconjugates specifically target tumor cells with overexpressed GLUT1 transporters. 4. Chimeric antigen receptor (CAR) T-cell therapy; CAR-T cells receptors recognize tumor antigens such as mesothelin, which triggers cytokine production and accumulation in the tumor region. Followed by regulatory T-cell (Treg), tumor-associated macrophage (TAM) and myeloid-derived suppressor cell (MDSC) infiltration, which eliminate the cancerous cells. TNBC cells express receptors, such as programed cell death protein (PD)-1, cytotoxic T lymphocyte-associated protein (CTLA)-4, and lymphocyte activation gene (LAG)-3, and derive immunosuppressive factors, like transforming growth factor-β (TGF-β), IL-10, and indoleamine 2,3-dioxyganase(IDO).

In this review, we focus on novel target therapies as an approach to revolutionize breast cancer treatment. We provide an update on therapies targeting components of the tumor microenvironment and finding the Achilles heel of hallmarks of cancer. By describing strategies to enhance efficacy while simultaneously limiting systemic toxicity, we want to underline the necessity of further TNBC-oriented research to provide patients with the utmost treatment. The uniqueness of this review lies in the detailed description of the core modifications that render healthy cells cancerous, underscoring the superiority of the malignant ones. We emphasize the critical points of metabolic and immune alterations, which can be targeted in order to maximize the impact of therapy on the tumor site. Not only do we point out cutting-edge methods such as glycoconjugates and CAR-T therapy, but by effectively communicating their distinctive features, including mechanisms, implications, and shortcomings, we aim to provide a better understanding of this matter. In Figure 1, we present the graphical abstract.

## 2. Deregulating Cellular Metabolism and the Warburg Effect

Cancer cells, in contrast to normal cells, are able to alter or reprogram cellular metabolism to meet the demands of the rapidly proliferating tumor [26]. The key difference between the cells in question is their distinct glucose metabolism. While healthy cells favor processing glucose under aerobic conditions through oxidative phosphorylation due to its effectiveness in ATP production, cancer cells develop in a hypoxic microenvironment. Thus, they induce mitochondrial metabolic changes, allowing them to produce energy through aerobic glycolysis [27]. This phenomenon is known as the Warburg effect, first described by Otto Warburg in 1920 [28]. 

The cornerstone of this modification is mutations in glycolytic genes, resulting in a strong preference for the fermentation of glucose to lactate even in the presence of sufficient amounts of oxygen [29]. It may seem that such a mechanism deprives cancer cells of growth-sustaining energy, yet nothing could be further from the truth. Admittedly, the catabolic efficiency of aerobic glycolysis is significantly lower than that of oxidative phosphorylation, as it produces a scarce 2 ATP molecules per glucose molecule [30]. However, reducing pyruvate to lactate instead of transporting it into the mitochondria provides neoplastic cells with a head start to unlimited proliferation by supplying them with building blocks such as nucleotides, amino acids, and lipids crucial for further development of the tumor [31]. 

To compensate for the small energy gain, cancer cells increase the rate of glucose. metabolism. Enhanced glucose uptake has its source in the overexpression of sugar transporters (GLUTs) [32]. It has been reported that among these transmembrane proteins, GLUT-1 is widespread in cancers such as hepatic, pancreatic, breast, esophageal, brain, renal, lung, cutaneous, colorectal, endometrial, ovarian, and cervical [33,34,35]. Therapeutically, GLUT-1 shows a critical advantage in terms of being a budget-friendly and minimally invasive prognostic marker. The more GLUTs 1 are expressed, the worse the prognosis in terms of treatment and survival [36,37]. GLUT-1 overexpression has also been linked to increased metastatic potential and aggressiveness of the tumor, allowing it to spread to adjacent tissues as well as the remote ones, thereby creating a new focus of the disease [38]. Given that the specified transmembrane transporter can bind not only to glucose but also to galactose, mannose, glucosamine, or ascorbic acid [39], insofar as sugar-binding drugs are considered, it is widely studied as a potential targeted therapy to battle cancer, which will be evaluated in this paragraph.

Another glucose carrier, the sodium–glucose cotransporter 1 (SGLT1), which sustains intracellular glucose levels regardless of the concentration of external glucose by utilizing sodium gradients, is overexpressed in a variety of cancer types and affects epidermal growth factor receptor (EGFR) activity to encourage TNBC cell proliferation [40,41,42,43]. This finding drew the attention of Satoko Tsunokake et al., who analyzed the expression levels of SGLT1 and SGLT2 in patients with breast carcinoma and assessed how SGLT1 inhibitors affect neoplastic cells in vitro. It is the first study demonstrating that SGLT1-specific inhibitors, such as mizagliflozin and KGA-2727, suppress breast carcinoma cell proliferation. Moreover, high SGLT1 expression proved to be a detrimental clinical prognostic factor in breast cancer patients. 

In recent years, another indispensable breast cancer metabolic feature has been emphasized, namely lipid metabolism. According to clinical evidence, obese postmenopausal women are 20–40% more likely than slender women to develop breast cancer [44]. To address the needs for membrane biosynthesis, generation of energy, and signaling molecule formation, tumor cells exhibit surged de novo fatty acid (FA) synthesis [45]. Its upregulation is attributed to the increased activity of fatty acid synthase (FASN), which makes use of acetyl-CoA and malonyl-CoA to create a saturated FA. While FASN is the key biosynthetic enzyme in the fatty acid synthesis pathway, the enzyme that sets the pace for fatty acid synthesis is acetyl-CoA carboxylase (ACC), which carboxylates acetyl-CoA to malonyl-CoA [46]. The progression of HER2-driven breast cancer is associated with FASN-positive rates, as evidenced by over 200 cases of metastatic breast cancer [47]. FA production is interestingly downregulated in TNBC in comparison to other BC subtypes [48]. Nonetheless, FASN inhibition has anticancer properties in TNBC cells that are both chemoresistant and sensitive, suggesting an indirect role for FASN in TNBC [49]. C75, a small-molecule inhibitor of type 1 mammalian FAS, showed significant antitumor effects against human cancer cell lines in vitro and against human breast. The initial in vivo evidence of tumor growth reduction following FASN inhibition by C75 notwithstanding, it also caused significant weight loss in mice, which constituted its dose-limiting toxicity [50]. Attention was then drawn to epigallocatechin-3-gallate (EGCG), the main polyphenolic catechin in green tea, which was reported to inhibit FASN, cause apoptosis in several tumor cell lines in vitro, and shrink tumor diameters in animal models [51,52,53]. Further in vivo studies are of great importance given that EGCG does not modulate carnitine palmitoyltransferase-1 (CPT-I) activity, which regulates the breakdown of fatty acids, thus evading the anorectic effects of FASN inhibition [54]. To emphasize the value of in vivo studies, combining EGCG with standard chemotherapeutic drugs has demonstrated tumor cell sensitization, enhancing treatment response in breast cancer [55]. 

The high rate of cell proliferation requires, in addition to an increase in demand for glucose and lipids, the reprograming of amino acid metabolism. The most prevalent amino acid in the blood is glutamine. Despite glutamine’s non-essential status, many malignancies, including TNBC, utilize it through seizing glutaminolysis—the process by which glutamine is catabolized for entry into the TCA cycle—to support energy production, glutathione (GSH) generation, and biosynthesis [48]. Two crucial transporters—L-type amino acid transporter 1 (LAT1) and alanine, serine, and cysteine-preferring transporter 2 (ASCT2)—are both overexpressed in TNBC [56]. Substantial ASCT2 expression is essential for glutamine intake and subsequent glutaminolysis [57]. Furthermore, a metabolomics study reveals that TNBC improves glutaminolysis despite its low glutamine and high glutamate levels [58]. The overexpression of glutaminase (GLS), the enzyme that generates glutamate from glutamine, makes TNBC more glutamine-reliant and sensitive to glutaminolysis-targeting therapies than other breast cancer subtypes [59]. Several small molecules have been discovered that inhibit GLS and GLS2 isoenzymes, namely CB-839, BPTES, and compound 968 [60]. Currently under development is a novel class of inhibitors, with the most advanced compound among them being UPGL00004, which shows better microsomal stability when compared with either BPTES or CB-839 [61]. Qingqiu Huang et al. examined a promising solution using UPGL00004 in a triple-negative breast cancer patient-derived tumor graft model by combining it with bevacizumab, an FDA-approved anti-angiogenesis monoclonal antibody that targets VEGF, administered via intraperitoneal injection. It resulted in an undetectable increase in tumor size during treatment. 

### 2.1. Glycoconjugates as a Trojan Horse Method to Deliver Drugs to Malignant Cells

Nowadays, research focuses on improving the properties of chemotherapeutics, as their well-known drawbacks, namely the lack of specificity in targeting the tumor cells within the neoplastic lesion and the increasing resistance of cancer cells, hinder their anticancer properties and result in systemic toxicity leading to detrimental side effects [62,63]. One of the possibilities is the aforementioned targeted therapy, which reduces the toxic effect on healthy cells by precisely delivering the biologically active compound to the pathologically altered region, thus facilitating the reduction in the therapeutic dose of the medication to attain an adequate intracellular concentration. The core of obtaining specificity is linking the therapeutic agent to an applicable ligand, functioning as a selective carrier tuned towards overexpressed receptors on malignant cells, to increase the probability of attached drug uptake [64]. In this review, we focus on glycoconjugates, molecules targeting the overexpressed GLUTs in breast cancer cells. Since cancer cells exhibit increased glucose metabolism, which has its reflection in the elevated number of glucose transporters, glycoconjugates can thus be selectively absorbed by pathological cells and scarcely affect healthy ones [65]. 

The study of glycoconjugates for GLUT1-targeted cancer therapy began in 1995 with the publication by Pohl et al. of glufosfamide. D-glucose was linked to ifosfamide’s alkylating moiety to boost the drug’s cancer-selective absorption, mediated by GLUT1. When glucose is hydrolyzed or cleaved by glucosidase, the active medication, isophosphoramide mustard, is released [66]. Over the past years, phase II trials of glufosfamide as a single agent in non-small-cell lung cancer (NSCLC), pancreatic adenocarcinoma (PC), and high-grade glioma (GBM) have been ongoing [67]. Furthermore, it has been observed that in breast carcinoma MCF-7 cells, ß-D-Glc-isophosphoramide mustard reduced DNA and protein synthesis and activated poly(ADP-ribose)polymerase [68]. Subsequently, glycoconjugates comprised of glucose attached to cytotoxic drugs such as adriamycin (ADM), oxaliplatin, paclitaxel, and methotrexate (MTX) are being developed regarding breast cancer therapy.

Adriamycin (ADM), also referred to as doxorubicin (DOX), is a DNA topoisomerase II inhibitor that is a member of the anthracycline anticancer medication family. By generating free radicals, it deals oxidative damage to DNA, inducing apoptosis [69]. DOX clinical uses are restricted due to several factors. Firstly, because of its dose-dependent cardiotoxicity. With doses of 400, 550, and 700 mg/m^2^, the patient’s predicted rate of heart failure was 5%, 26%, and 48%, respectively [70]. Alopecia, stomatitis, myelosuppression, severe nausea and vomiting, and extravasation are other prevalent toxicities associated with DOX use [71]. Secondly, the emerging multidrug resistance contributes to unsatisfactory therapy outcomes and poor prognosis. The tumor microenvironment and restricted vascular accessibility are recognized as noncellular resistance [72]. The cellular resistance mechanism is connected to ATP-binding cassette membrane transporters, operating as ATP-dependent efflux pumps, pumping out chemotherapeutic agents out of cancerous cells [73,74]. Non-transport-based cellular resistance is a result of modifications in particular enzyme activity and cell death programs, specifically autophagy [75] and apoptosis [76]. 

A potential strategy to improve ADM efficiency and selectivity is, by using covalent conjugation, to attach the drug to a targeting ligand that can bind to tumor cells with high specificity and generate an ADM prodrug [77]. The primary amino group of the ADM molecule, which is located far from the anticancer active anthracene ring, has the highest reactivity, according to the drug design concept. More importantly, altering the primary amino group with other molecules will not impact ADM’s antitumor activity. Therefore, it is viable to create a durable amide bond by covalently binding the amino group to the carboxyl groups. Furthermore, research has demonstrated that altering the primary amino group may significantly lower cardiotoxicity, which is one of the key goals [78]. Research indicates that 2-amino-2-deoxy-d-glucose (2DG) is a glucose analog that is recognized and transported into cells by GLUT1 on the cell membrane. It is formed by hexokinase phosphorylation of 2-deoxyglucose-6-phosphate [79,80,81]. 

Based on these findings, Cao et al. conjugated adriamycin with 2-amino-2-deoxy-D glucose and succinic acid (2DG-SUC-ADM), designed to target tumor cells through GLUT1. The primary amin group was modified with 2DG using succinic anhydride as the linker between the ligand and the therapeutic payload. The study was conducted on five tumor cell lines: MCF-7 (luminal A molecular subtype breast cancer), Bel-7402 (hepatocellular 261 cancer), HepG2 (hepatocellular cancer), MDA-MB-231 (TNBC), U87MG (glioblastoma), and 262 SKOV3 (ovarian cancer). The HELF cell line was used as normal cells. After incubating MCF-7 263 cells in 2DG-SUC-ADM or in free ADM, the results showed elevated levels of 2DG-SUC-ADM 264 in the cytoplasm, whereas levels of free ADM were very low. To support the assumption that the in vitro tumor-targeting ability of 2DG-SUC-ADM is mediated by GLUT1, In vitro blocking and inhibition were performed using 2DG or quercetin. The results showed successful inhibition of 2DG-SUC-ADM uptake, confirming the uptake of 2DG-SUC-ADM was through GLUTs. As for in vitro antitumor activity, 2DG-SUC-ADM effectively reduced the viability of cancer cells (MDA-MB-231 and HepG-2) while having little cytotoxicity in normal cells (HELF) compared with free ADM. Moreover, 2DG-SUC-ADM showed high antitumor efficacy on ADM-resistant MCF-7 cells (MCF-7/ADR cells) compared to free ADM treatment. In vivo studies were conducted on mice bearing S180 and SKOV3 tumors. Not only did 2DG-SUC-ADM inhibit tumor growth with a rate of 64.6% and 68.8%, respectively, but it also barely reduced the SKOV3-bearing mice body weight and interestingly increased the body weight of mice-bearing S180 tumors. Compared to the free ADM group, the hearts and kidneys of the mice injected with 2DG-SUC-ADM did not exhibit any discernible pathologic alterations. 

Strong broad-spectrum antitumor medications, known as platinum-based anticancer drugs (PBDs), are effective against a variety of solid tumors, including breast cancer. US Food and Drug Administration (FDA) approval for cisplatin [cis-diammine(dichloro)platinum(II)], a platinum-based compound, was given in the late 1970s [82,83]. Hundreds of PBDs have been developed to date and underwent clinical trials with the aim of outperforming cisplatin in terms of anticancer activity while minimizing side effects such as nephrotoxicity, cardiotoxicity, hepatotoxicity, neurotoxicity, and resistance [84,85]. The FDA has approved only oxaliplatin and carboplatin for the treatment of cancer [86,87]. For the treatment of metastatic breast cancer, oxaliplatin, a third-generation platinum derivative, has taken the place of cisplatin as a more effective and secure medication [88]. Through the segregation of transcription factors, oxaliplatin produces intrastrand and interstrand DNA–platinum adducts that disrupt gene transcription and/or result in G2/M stage arrest [89]. However, oxaliplatin has several drawbacks, including acute dysesthesia and peripheral distal neurotoxicity [90]. Moreover, oxaliplatin treatment is associated with severe and dose-limiting sensory neuropathy in addition to gastrointestinal and renal adverse effects, as well as potentially fatal thrombocytopenia [91,92]. 

Liu et al. developed three oxaliplatin-based sugar conjugates: glycosylated (trans-R, R-cyclohexane-1,2-diamine)-malonatoplatinum(II) complexes. Compared to oxaliplatin, the sugar conjugates showed a notable improvement in water solubility. Measurements were made of the cytotoxicity against six human cancer cell lines, including MCF-7 cells. Following 96 h of complex exposure, the dose dependence of the surviving cells was used to calculate the IC50 values (indicating how much of a drug is needed to inhibit a biological process by half). Among all the studied cell lines, compound 3 (fluro-substituted), which is the most water soluble, exhibited the strongest activity. When applied to MCF-7 breast cancer cell lines, Complex 3 was found to be twice as cytotoxic as the therapeutic medication oxaliplatin. An acute toxicity study using immunodeficient BALB/c nude mice was carried out to evaluate the potential safety of the sugar conjugates. The lethal dosage values (LD50) and maximum tolerated dose (MTD) of complexes 2 and 3 were almost 5-fold higher than those of oxaliplatin, suggesting that the Pt(II) complexes’ increased water solubility may have significantly improved the viability and possible safety of high-dose treatment. 

One of the most popular and efficient therapeutic drugs for treating a variety of malignancies, including breast cancer, is methotrexate (MTX) [93]. However, due to its lack of tumor specificity, MTX has several drawbacks, such as nephrotoxicity and neurotoxicity [94,95]. The primary metabolite of MTX, 7-OH-MTX, is less soluble in water and may exacerbate kidney damage when taken in large dosages [96]. MTX’s weak pharmacokinetic characteristics can lead to an inadequate clinical response. While increasing the dosage of MTX enhances therapeutic efficacy, it also raises the chance of side effects [97]. In general, life-threatening toxicity rather than MTX’s lack of efficacy is the main cause of therapy cessation. When administered at low doses, MTX’s bioavailability is around 100%; however, at high doses, it drops to a scarce 10% to 20%. Shortly after administration, the kidneys remove a significant portion of the MTX, causing the medication concentration in the target tissues—solid tumors—to decrease rapidly [98]. 

Woźniak et al. designed a next-generation tumor-targeting MTX delivery system for enhanced selectivity and decreased systemic toxicity. The synthesized compound is a novel glucose-methotrexate conjugate (GLU-MTX), in which MTX, D-Glucose, and the linker are connected via a cleavable linkage susceptible to the action of hydrolytic enzymes. In vivo studies were conducted on five human cancer cell lines, including MCF-7. The results showed that, with IC50 values comparable to those of free MTX, GLU-MTX demonstrates strong anticancer efficacy against a variety of solid tumor cell lines. Furthermore, GLU-MTX selectively destroyed cancer cells while posing little threat to non-cancerous cells (the healthy fibroblast WI-38 cell line). It was proven that the cellular uptake of GLU-MTX is glucose transporter-specific, which had its reflection in the uptake of GLU-MTX in cancer cells—it was 17-fold more efficient than that of MTX. In vitro studies in breast tumor-bearing mice found that Glu-MTX caused a significant tumor growth delay compared to MTX-treated and control mice.

### 2.2. Shell-Shedable Micelles as Yet Another Drug Delivery System

Enhancing drug selectivity to reduce systemic toxicity while simultaneously overcoming the tumors’ unfavorable environment remains the crucial challenge in targeted therapy. Domiński et al. developed micelles encapsulating glycoconjugated drugs, given that the tumor-specific Warburg effect is utilized by both vectors. Micelles, as self-assembled nanostructures, possess hydrophilic and hydrophobic parts. The outside hydrophilic shell protects the nanocarrier from being recognized by the immune system and, consequently, from being removed by macrophages. On the other hand, delicate hydrophobic anticancer treatments may be contained within the hydrophobic core [99]. Thereby, encapsulated anticancer drugs have a greater retention effect and increased permeability, which promote drug accumulation in tumor tissues. As a result, the drug’s therapeutic dose can be decreased, reducing negative side effects [100]. Among the various available biodegradable nanomaterials, poly(3-hydroxybutyrate) (PHB) has a high hydrophobicity, thus making the nanoparticles more stable and resistant to biodegradation [101]. Furthermore, PHB breaks down and resorbs in vivo, which means that its breakdown products are removed naturally by filtration and post-metabolization without being detrimental to cells [102]. 

The Warburg effect, due to an increased glycolysis rate leading to pyruvate accumulation, which is further converted into lactic acid, induces an acidic microenvironment (pH 6.5–6.8) [103]. Thus, studies focus on pH-sensitive drug carrier development to further improve cancer targeting and selectivity [104,105]. Acid-sensitive chemical linkages, which are rapidly hydrolyzed in an acidic environment but stable at physiological pH, provide the basis for pH-responsive nanocarrier creation [106]. Of these, the hydrazone linkage has garnered significant interest in anticancer drug delivery systems owing to its rapid hydrolysis in a mildly acidic environment and stability at physiological pH [107]. By adding acid-labile links to the polymer structure, nanocarriers can be modified to disassemble and alter their size, shape, and surface charge in response to the acidic tumor microenvironment [108]. Consequently, the medications that are encapsulated are delivered in controlled quantities and can specifically target tumor locations. Lastly, to render the surface of the micelles imperceptible, prevent undesired interactions with proteins, and lengthen the time that nanocarriers spend in the bloodstream, PEGylation is carried out. 

All in all, micelles developed by Domiński et al. comprised poly(ethylene glycol) and poly([R, S]-3-hydroxybutyrate) blocks linked via a hydrazone bond. The resultant amphiphilic copolymer can self-assemble into nanoscale, pH-triggered, shell-sheddable micelles, which were used to deliver encapsulated anticancer drugs (doxorubicin and 8-hydroxyquinoline glycoconjugates). Under physiological conditions (pH 7.4), the micelles were stable, whereas doxorubicin was released when the hydrazone connection was dissolved by acid, causing the PEG chains to shed. Studies on in vitro drug release showed that after 8 h, the drug release was 21%, 37%, and 47% at pH 7.4, 6.4, and 5.5, respectively, indicating pH-dependent drug release behavior. In vitro cytotoxicity was first evaluated with blank micelles tested on HCT-116 (colorectal carcinoma cell line) and MCF-7 (human breast adenocarcinoma cell line), along with a healthy cell line, NHDF-Neo (Normal Human Dermal Fibroblasts-Neonatal). The results showed no toxic impact on all tested cell lines, confirming their safety for healthy cells. The cytotoxic activity of drug-loaded micelles (DOX-micelles, 8HQ-mic, 8HQ-Glu-mic, and 8HQ-Gal-mic) was significantly higher compared to free drugs, exhibiting strong effects at low doses. While the tested glycoconjugates (8HQ-Glu and 8HQ-Gal) turned out to be toxic to healthy cells (NHDF-Neo), 8HQ-Glu-mic and 8HQ-Gal-mic gained a higher selectivity index towards MCF-7 cells (SI = 22.68 and 14.68, respectively). 

### 2.3. Rg3 Liposomes Interacting with GLUT-1

One of the most effective anticancer drugs used in clinical practice today is paclitaxel, a diterpenoid taxane derivative [109]. It functions through a specific process that involves binding tubulin to stabilize the development of microtubules, which ultimately disturbs mitosis and results in cell death [110,111]. Paclitaxel’s therapeutic value is, however, limited by certain disadvantages. For instance, it does not exhibit specific cytotoxicity toward cancer cells. Thus, the drug’s activity has an impact on both healthy and malignant cells, leading to adverse side effects [112]. Paclitaxel’s poor water solubility is another issue that significantly limits the scope of its therapeutic use [113]. Lastly, multidrug-resistant (MDR) breast cancer chemotherapy faces a significant obstacle in the form of PTX resistance, either inherited or acquired. 

Zhu et al. studied whether functional Rg3-based liposomes could carry PTX into tumors efficiently, target and remodel the TME to reverse the MDR condition and enhance the therapeutic efficacy of PTX treatment by dual targeting of the tumor microenvironment and cancer cells. (R)-Rg3, a monomer extracted from ginseng, has been reported to exhibit anticancer properties, such as inducing tumor cell apoptosis, inhibiting tumor invasion, proliferation, and angiogenesis, and reducing metastasis and recurrence [114,115]. According to a recent study, Rg3 can reverse the MDR of several tumors in vitro [116,117]. In addition to its hydrophilic domain, which contains two glucosyl groups, Rg3 additionally includes a hydrophobic region with a steroid structure similar to cholesterol, a necessary component of liposomes. It was anticipated that Rg3 might not only be substituted for cholesterol as liposomal membrane material but also boost the uptake efficiency of liposomes by tumor cells through its identification by GLUT-1. Prior research has indicated that Rg3 may function as a liposomal membrane material, with its glucosyl side chains capable of binding to GLUT-1’s preferable amino acid residues [118,119]. Firstly, confocal microscopy and flow cytometry, using MCF-7/T cells and coumarin-6-labeled liposomes, showed that the fluorescence intensity in MCF-7/T cells treated with Rg3-liposomes (Rg3-409 LPs) was twice as high as in cells treated with cholesterol-liposomes (C-LPs). After results revealed that Rg3 as the membrane material of the functional ginsenoside Rg3 liposomes enhanced liposome uptake efficiency and penetration in spheroids, the combination with paclitaxel (Rg3-PTX-LPs) was evaluated. MCF-7/T cells were not sensitive to PTX treatment alone, while PTX/Rg3 combination therapy induced cytotoxicity in MCF-7/T cells with a 2-fold lower IC50. For the PTX, PTX/Rg3, C-PTX-LPs, and Rg3-PTX-LPs groups, the proportion of apoptotic cells depicted in flow cytometry was 11.95%, 30.20%, 28.65%, and 51.73%, respectively. Lastly, in vivo studies were consistent with in vitro spheroid penetration results. Moreover, none of the treatment groups showed any obvious pathological changes, and there was no discernible variation in body weight across the groups. Overall, this study indicates that the ginsenoside Rg3 liposome delivery system enhances both safety and tumor targeting in mammalian cancer conditions.

## 3. HIF-1 at the Crossroads of Acidosis and Hypoxia

The Warburg effect has long-range consequences that alter the tumor microenvironment. Rapid growth along with excessive lactate production cause, respectively, an exceedance of their blood supply and acidification of their microenvironment. The tissue becomes short of oxygen and nutrients and has a low pH. Under such conditions, prolyl hydroxylase domain protein 2 (PHD2) becomes inactive, and HIF1 escapes ubiquitin degradation [120,121], which allows it to regulate the expression of VEGF, TGF-ß, PDGF-B, PAI-1, and EPO genes [122]. The listed factors partake in angiogenesis, resulting in the formation of atypical vessels with flaws such as restricted blood flow, excessive permeability, and chaotic structure [123]. The adverse HIF-1 activation can also be hypoxia-independent in cancer cells. Loss of the tumor suppressor gene p53 results in HIF-1 overexpression, as p53 promotes MDM2-mediated ubiquitination of HIF-1, promoting metastasis and resistance to apoptosis [124]. Acidosis, hypoxia, and HIF-1 are inextricably connected. Hypoxia triggers HIF-1 activation, which increases the expression of glucose transporters and glycolytic enzymes such as lactate dehydrogenase, responsible for turning pyruvate into lactate, to enhance glycolysis [125]. Interestingly, tumor cells create a symbiotic interaction. In the tumor, the further from the blood vessels, the lower the pH [126]. The tumor’s hypoxic cancer cells, located in the middle of the tumor, produce lactate, which is reproduced into pyruvate by aerobic cells during oxidative phosphorylation. Not only does it ensure efficient glucose utilization, but it also protects cancerous cells from rapid intracellular pH drops, which would cause apoptosis. The key factor responsible for eliminating lactate from hypoxic cells is the HIF-inducible plasma membrane monocarboxylate transporter 4 (MCT4) [127]. Tumor cells’ intracellular pH (pHi) stays between 7.0 and 7.4, despite the extracellular pH having the potential to drop from 7.4 to 6.0. Carbonic anhydrases (CAs), which catalyze the reversible conversion of CO_2_ and H_2_O to HCO_3_ and H+, are expressed by all cells. Expression of the transmembrane CAs, CA9 and CA12, is upregulated with HIF-1 activation [128]. The extracellular environment becomes more acidic due to H+, but internal pH can be raised by recycling HCO_3_. Since CA9 expression is found solely in malignant epithelium and is elevated in pre-invasive DCIS of the breast, it is linked to a poor prognosis in breast cancer [129]. Therapeutically, hypoxia and acidosis contribute to both radio resistance and chemoresistance. The effectiveness of radiation therapy depends on the oxygen supply, as the core of organic peroxide formation sets off DNA damage. The oxygen-deprived microenvironment allows target molecules to decondition the adverse impact, retrieve, and resume normal function [126]. HIF-1 promotes P-glycoprotein expression, also known as multidrug resistance protein 1, which operates as an ATP-dependent efflux pump, pumping out chemotherapeutic agents from cancerous cells, making chemotherapy inefficient [130]. 

### NDBT Inhibition as an Approach to Reduce Acidosis and Hypoxia in TNBC

As mentioned above, significant upregulation of CA9 and CA12 occurs in hypoxia, depending on HIF1. To control intracellular pH (pHi), CA9 cooperates with Na+-driven bicarbonate transporters (NDBTs), the loss of NDBTs causes a decrease in CA9’s enzymatic effectiveness [131]. Na+ and HCO_3_ are co-transported into cells by NDBTs. The NDBT family has several members, with SLC4A7 attracting the most attention owing to its highest expression in breast cancer [132]. Studies have proven that along with increased CA9 expression comes NDBT overexpression [133,134]. Approximately 90% of mortality from breast cancer is related to metastasis, which is particularly prevalent in areas with hypoxia and acidosis [135]. Lysyl oxidase (LOX), matrix metalloproteinases (MMPs), and genes that promote epithelial-mesenchymal transition (EMT) are among the genes whose expression is regulated by hypoxia and HIF at every phase of the metastatic process [136]. Mesenchymal phenotype and motility are acquired by epithelial cells via a series of biological events that culminate in the complex process known as the epithelial-to-mesenchymal transition (EMT). Cell adhesions are lost during carcinogenesis, along with alterations in cell and cytoskeleton polarization, detachment, migration, intravasation, survival in the vascular system, extravasation, and ultimately metastasis [137]. E-cadherin expression is downregulated during EMT, while vimentin and N-cadherin levels are elevated [138]. Interestingly, only E0771 tumor-bearing mice fully resisted tumor recurrence, which may be attributed to varying amounts of natural immune cells, indicating the importance of further evaluation of the myeloid-derived suppressor cells (MDSCs) and CD8+ and CD4+ cells role in the anti-tumor response. Compared to other breast cancer subtypes, TNBC is more often hypoxic and has a greater incidence of metastasis [139], hence, developing targeted therapeutics to prevent hypoxia and acidosis-induced metastasis should be put forward to increase overall patient survival.

Targeting HIF-1 is crucial for treating TNBC since it enhances and activates breast cancer stem cells and promotes glycolysis, metastasis, angiogenesis, and immune evasion. Thus far, several agents inhibiting the activation of HIF-1 in TNBC cell lines are in the preclinical phase, which has been mustered by Liu et al. [140]. 

McIntyre et al. studied S0859, a drug inhibiting sodium-dependent bicarbonate co-transporters, and its impact on intracellular pH regulation and tumor growth suppression. SLC4A9-targeting shRNA was also evaluated. MDA-MB-468, MDA-MB-231, MCF7 cell lines were used in vitro. Spheroids were initiated through aggregation of these cells. The results showed SLC4A9 knockdown significantly reduced hypoxic induction of SLC4A9 expression and reduced the spheroid growth rate in MDA-MB-231 by 39% at 72 h normoxia or hypoxia (0.1% oxygen). As for S0859 (100 μmol/L), by inhibiting sodium-driven bicarbonate transporters, it reduced spheroid growth in MDA-MB-231 by 48% during an 11-day period. Either SLC4A9 knockdown or S0859 treatment in MDA-MB-231 acidifies the pH of cells in spheroids, with the strongest effect at the spheroid core (pH difference −0.14 at the core for SLC4A9 knockdown, −0.19 for S0859 treatment). Lastly, both knockdown and inhibition of sodium-driven bicarbonate transporters enhanced apoptosis in the core of MDA-MB-231 spheroids. The apoptosis marker-cleaved caspase-3-was stained on spheroids treated with S0859 or NDBT knockdown and immunohistochemistry was carried out. In vivo study using SLC4A9 knockdown reduced the growth rate of MDA-MB-231 orthotopic xenografts by 79%.

Carroll et al. focused on scrutinizing the role of NDBT in metastasis in four TNBC cell lines (HCC-1806, MDA-MB-231, CAL-51, and SUM159PT). Lentiviral shRNA was used to knockdown SLC4A4 and SLC4A5 in TNBC cell lines MDA-MB-231. The results were validated by Western blot in normoxia and hypoxia. NDBT inhibition by S0859 lead to reduced migration (assessed using the wound healing assay) and invasion (assessed using the modified Boyden chamber assay) in all four cell lines investigated in hypoxia (1% O_2_), respectively 15–50% and 50–90%. EMT was also the focus of the study due to its crucial role in changing morphology and triggering invasion and migration. The expression of important EMT genes, such as Twist, ZEB1, Snail, and Goosecoid, was decreased by NDBT knockdown. In addition, the NDBT knockdown in HCC1806 resulted in a reduction in the mesenchymal marker vimentin and a surge in the epithelial marker E-cadherin. The prevalent metastatic location of TNBC, the lung, was substantially less invaded by TNBC after SLC4A4 and SLC4A5 were knocked down in vivo, respectively by 94% and 98% over a 50-day course. 

Nief et al. introduced “BiCyclA”, which is an innovative approach aiming to generate an anti-tumor response without the need for checkpoint inhibitors (which remain low for TNBC patients) by inhibiting immunosuppression. BiCyclA stands for bicarbonate to decrease the tumor’s acidity, cyclophosphamide to diminish Tregs, and ablation to elicit an anti-tumor immune response. It has been demonstrated that tumor ablation, which involves destroying a tumor with brief, harsh conditions (heat, cold, or chemicals), triggers an immune response against the tumor by releasing tumor-associated antigens (TAAs) after tumor necrosis [141]. In vivo studies were carried out on mice bearing 67NR, 4T1-Luc, or E0771 tumors, representing three different TNBC immunophenotypes. The majority of mice with 4T1-Luc, 67NR, and E0771 tumors were cured by BiCyclA (sodium bicarbonate (200 mM p.o.), cyclophosphamide (100–200 mg/kg i.p.)), respectively 50%, 60% and 70% 50 days after treatment. 

## 4. Role of LOXL2 in Triggering Metastasis in TNBC

As indicated above, TNBC is considered to be the most aggressive breast cancer type, owing to its unfavorable hypoxic microenvironment, hormonal therapy insensitivity, high recurrence rate, and significant metastatic potential. The subpopulation of cells known as breast cancer stem cells (CSCs) that dwell inside the tumor and possess the ability to initiate tumorigenesis is responsible for tumor relapse [142]. The concept behind CSCs, which postulates that just a fraction of the tumor’s cells is responsible for cellular heterogeneity and tumor maintenance, has explained their formation [143]. According to the CSC theory, mesenchymal and apical–basal polarity are often lost in epithelial cancer cells, which results in a more invasive and metastatic phenotype and the eventual formation of CSCs. Epithelial-mesenchymal transition (EMT) is the term used to describe this phenomenon [144]. LOXL2, a member of the lysyl oxidase (LOX) family, is a copper-dependent oxidase primarily responsible for catalyzing the covalent cross-linking of collagen and elastin inside the extracellular matrix (ECM) [145].

Furthermore, several studies have shown that LOXL2 can trigger epithelial-mesenchymal transition (EMT) and can act as a tumor suppressor or promoter of metastasis [146,147]. Importantly, there have been reports of increased LOXL2 expression in breast cancer cells [148,149], and especially in TNBC cells [150]. In prospective research with patients, LOXL2 expression was found in 16.2% of patients. In comparison to non-TNBC patients, patients with triple-negative breast cancer (TNBC) exhibited a statistically significant 12.3% higher rate of LOXL2-positive expression [151]. LOXL2 overexpression is not only associated with tumor progression and metastasis but is also an independent prognostic marker in breast cancer patients [152]. Hypoxic conditions generated in the tumor microenvironment are favorable to LOXL2 overexpression, as the activated HIF-1 binds to the hypoxia-responsive element (HRE) in the promoter region of the LOXL2 gene [153]. The deamination of lysine residues mediates the ECM’s collagen cross-linking by extracellular LOXL2, which enhances the ECM’s rigidity. Through integrin activity modulation, focal adhesion formation, and signaling, this process facilitates tumor cell invasion and progression [147]. Through modulating the deposition of collagen in the vascular milieu, LOXL2 plays a role in vessel development, endothelial cell proliferation, and migration [154]. Both the enzymatic and non-enzymatic functions of LOXL2 are required for its role in the activation of angiogenesis. The expression levels of LOXL2 determine how endothelial cells are arranged into tubes. However, enzymatic activity is necessary for the balance of the vasculature and basement membrane structures [155]. As an essential pro-lymphangiogenic protein, LOXL2 influences lymphatic endothelial cells (LEC) in vivo and in vitro functions. Breast cancer patients’ survival rates are correlated with the lymph angiogenesis process, which is essential for the malignancy of the disease [156].

### Evolution of LOX Enzyme Inhibitors 

The first ever reported LOX inhibitor, aminopropionitrile (BAPN), is present in literature dating back to the 1950s. It is an irreversible, non-specific LOX inhibitor that drastically impairs collagen and elastin cross-linking [157,158]. Rats with 7,12-dimethylbenzanthracene-induced breast tumors were studied by Cohen et al. Researchers concluded that BAPN caused an 82% reduction in tumor development and a considerable reduction in tumor volume, in addition to inhibiting the collagen cross-link. In a different study, mice were injected with MDA-MB-231-Luc2 breast cancer cells to examine the impact of BAPN on organ invasion [159]. The findings indicate that BAPN lowered the incidence of metastases. When BAPN treatment was started the day before or the same day as the intracardiac injection of cancer cells, the number of metastases was reduced by 44% and 27%, respectively. On the other hand, BAPN did not affect the metastases that were already developed. Rachman-Tzemah et al., using a mouse model of breast cancer and LOX pharmacological suppression with BAPN or an anti-LOX antibody before surgical intervention, proved to decrease lung metastases following surgery and increase animal survival. Such intriguing results notwithstanding, BAPN does not have enough sites for chemical changes. This characteristic renders preclinical optimization limited [160].

In contrast, this disadvantage does not apply to novel types of LOX enzyme inhibitors, which are beneficial in the drug discovery process. Despite strong supporting data in previous cellular studies [161], clinical trials with the promising humanized form of the AB0023 antibody (simtuzumab) have not yet been successful in treating pancreatic, colorectal, or breast carcinomas (as well as fibrotic disorders) [162]. The fact that simtuzumab only targets extracellular LOXL2 may have contributed to this result, indicating that LOXL2 functions through intracellular pathways in metastatic progression [163]. Further studies identified the location of LOXL2 alterations in the pathological condition. These findings verified that LOXL2 is localized in the cytoplasm of breast cancer tissues, while in normal tissue, it surrounds the membrane [164]. 

Chang et al. evaluated the efficacy of two novel LOXL2 inhibitors, PXA-S1A and PXA-S2A, along with its oral pro-drug known as PXS-S2B. The modification of PXS-S1A led to the development and generation of PXS-S2A. When tested against the native human LOX enzyme and the recombinant LOXL2 enzyme, PXS-S1A, which is admittedly as active and selective as BAPN, shows practically equal results. Nonetheless, the key distinction between the two is that the former species exhibits more selectivity due to its chemical tractability, enabling structural modifications. After oral gavage, PXS-S2B is quickly absorbed, distributes evenly among tissues, and forms PXS-S2A, which exhibits remarkable in vitro characteristics such as high plasma stability, low plasma protein binding, and high metabolic stability. A tolerable dose of 10 mg/kg was found after PXS-S2B was dosed daily for 24 weeks in healthy mice. In vitro proliferation, migration, and invasion experiments using the MDA-MB-231 triple-negative human breast cancer model were used to evaluate the effectiveness of these LOXL2 inhibitors. Over the course of eight days, the dual inhibitor PXS-S1A and the LOXL2-specific PXS-S2A both suppressed cellular proliferation in dose-dependent ways. The results showed that LOX and LOXL2 are critical for the growth of breast cancer cells. These findings were validated in cells treated with shLOXL2, which showed reduced cell proliferation in comparison to the control group (scrambled control cells). It was demonstrated that PXS-S2B reduced primary tumor growth by around 55%, whereas PXS-S1A reduced tumor growth by approximately 75%. According to a press release, these LOXL2 inhibitors successfully concluded phase I clinical trials, showed favorable outcomes at a variety of doses, and sustained the intended level of inhibition for a full day. The trial’s underlying data have not yet been made public [165]. Metastasis in the liver and lung was significantly reduced by inhibiting both LOX and LOXL2, but this was not observed when LOXL2 was inhibited alone. Nevertheless, there was a decrease in vessel density following PXS-S2A treatment, which restricted the blood supply to the primary tumor. By suppressing LOXL2-directed early angiogenesis, the growth of the primary tumor was inhibited. 

De Vita et al., with the least amount of systemic toxicity possible, created a lipid-based vesicle that targets LOX in the tumor ECM and selectively concentrates epirubicin (EPI) at the tumor site. Compared to other examined formulations, Lipo-EPI-LOX demonstrated greater internalization and cytotoxic activity in in vitro studies on MDA-MB-231 cells. To compare Lipo-EPI-LOX internalization to the standard treatment groups of EPI and Lipo-EPI, functional in vitro assays were conducted. Comparing Lipo-EPI-LOX particles to free EPI and Lipo-EPI, confocal analysis showed a significant increase in EPI delivery at both time points (i.e., 6 and 48 h). This was followed by the cytotoxic effect of Lipo-EPI-LOX on the viability of the MDA-MB-231 assessment. While Lipo and Lipo-LOX showed no discernible impact on survival, Lipo-EPI and Free EPI demonstrated ˂80% of cell death, and Lipo-EPI-LOX demonstrated a reduction in cell viability with more than 80% cell death. Furthermore, in vivo tests demonstrated that Lipo-EPI-LOX had increased therapeutic activity, allowing a notable rise in the number of mice that survived. An orthotopic xenograft mouse model of human TNBC was used to assess the study. The mammary fat pad of NU/NU nude mice was subcutaneously injected with MDA-MB-231, which had been genetically altered to express firefly luciferase. Bioluminescent imaging (BLI) and fluorescence imaging were used to measure the distribution of tumor cells and EPI, respectively at 0, 1, 2, and 24 h in the biodistribution analysis. Results showed that Lipo-EPI-LOX formulation delivered Epirubicin to TNBC tumors most efficiently. The TNBC tumor model was utilized to investigate the therapeutic potential of Lipo-EPI-LOX. When the tumors reached a mean volume of 250–300 mm^3^,mice were divided into groups and received a weekly intravenous injection of empty liposomes (control group), Lipo-LOX, Lipo-EPI, Free-EPI, and Lipo-EPI-LOX over 33 days; injections of drugs 3.24 mg/kg. Compared to other treatments, treatment with Lipo-EPI-LOX significantly inhibited tumor growth. It was found that the Lipo-EPI-LOX treatment demonstrated >75% survival at the end of the survival study, which occurred 98 days after inoculation. Lipo-EPI-LOX further showed a decrease in systemic toxicity, based on total body weight gain (6%) and the absence of significant inflammatory infiltration of myocardial tissues. 

## 5. TILs-Key Contributors in TNBC’s Immune Evasion Strategy 

Strong perceptions of breast cancer’s poor immunogenicity are fueled by the fact that a majority of them are expert manipulators and evaders of immune destruction. Not only do they express immune inhibitory co-stimulatory receptors, such as programed cell death protein (PD)-1, cytotoxic T lymphocyte-associated protein (CTLA)-4, and lymphocyte activation gene (LAG)-3, but they also derive immunosuppressive factors, like TGF, IL-10, and IDO. Among the best-characterized mechanisms describing breast cancer’s ability to elude immune destruction is also the infiltration of suppressive immune cells, regulatory T cells (Tregs), myeloid-derived suppressor cells (MDSCs), and tumor-associated macrophages (TAMs) in the microenvironment [166]. Furthermore, it has been demonstrated that human BC cells can improve self-tolerance by avoiding and changing NK cell function [167]. The immunogenicity and immune cell infiltration of TNBC and HER2-amplified breast cancer are higher. Studies using modern chemotherapy to assess tumor-infiltrating lymphocytes (TILs) have confirmed that TILs are predominantly detected in highly proliferative tumors (TNBC and HER2-positive breast cancers) and that their presence at diagnosis is linked to both disease-free (DFS) and overall survival (OS) following adjuvant chemotherapy in these subtypes [168,169]. It has been determined that TILs in breast cancer are a population of lymphocytes that mostly consists of cytotoxic (CD8+) T cells, along with varied amounts of helper (CD4+) T cells, CD19+ B cells, and uncommon NK cells [170]. Tumor-infiltrating lymphocytes (TILs) with cytotoxic CD8+ and regulatory FOXP3+ expression, microsatellite instability, high tumor mutational burden, and the expression of immune checkpoint molecules like programed death ligand 1 (PD-L1) are some of the main factors that contribute to TNBC’s immunogenicity [171]. An elevated mutational burden probably corresponds with an increased likelihood of containing immunogenic neoantigens. Numerous research works have detailed T-cell responsiveness to neoantigens specific for malignancy [172,173]. Tumor-specific antigens called neoantigens are produced when somatic DNA undergoes mutations. Neoantigens typically exhibit a highly anticipated affinity for binding to MHC molecules. The consequences of mutational burden were investigated in a meta-analysis of six tumor types, including breast cancer, and it was shown that neoantigens correlate with enhanced overall survival independent of clinicopathological prognostic factors [174]. It was noted that patients with higher neoantigen loads also expressed more of the immunological checkpoints CTLA4 and PD1, suggesting that these patients would be the most suitable candidates for immune-checkpoint blockage [175]. It was also assessed how CD8+ and CD4+ T lymphocytes identify distinct tumor antigens [176]. 

### 5.1. CAR-T Therapy Generations

One kind of immunotherapy derived from adoptive T-cell transfer (ACT) is chimeric antigen receptor (CAR) T-cell treatment [177]. T cells from the patient are isolated from autologous peripheral blood and then modified ex vivo to express artificial receptors that identify tumor-associated antigens (TAAs). CAR-T cells are then cultivated ex vivo for amplification before being reinfused into patients as a cancer-fighting therapy [178]. A CAR’s four main segments are as follows: an extracellular domain that typically contains a single-chain variable fragment (scFv) derived from the variable region of antibodies for tumor antigen recognition; an extracellular spacer that controls the distance between tumor cells and CAR-T cells; a transmembrane domain that attaches the synthetic CARs to the patient’s T-cell membrane; and an intracellular signaling domain that includes CD3 and costimulatory domains for T-cell activation. CAR-T cells identify antigens on the surface of tumor cells when they come into contact with them [179]. Because the CAR-T cells exhibited low persistence and failed to expand, the initial generation of CAR- T-cell treatment produced unsatisfactory clinical results [180,181]. Costimulatory signaling domains were added to CARs during additional engineering to address these problems. To increase the retention period, the second generation of CARs adds one costimulatory domain (such as CD28, 41BB, or ICOS) to the CARs in comparison to the first generation [182]. To improve T-cell persistence and cytocidal potential, the third generation of CARs incorporates two additional costimulatory domains (e.g., CD27, CD28, 41BB, ICOS, and OX-40) [183]. The fourth generation of CARs, referred to as T-cells redirected for universal cytokine-mediated killing (TRUCKs), incorporates an inducible IL-12 cassette into the nuclear factor of activated T cells (NFAT) domain [184,185]. In this generation, upon recognition of tumor antigens by CAR-T cells and activation of the downstream signaling pathways, the pro-inflammatory cytokine IL-12 is produced and accumulates in the targeted region. The innate immune system, which includes macrophages and NK cells, is then drawn to tumors to alter the tumor microenvironment and eliminate the cancerous cells [186]. Currently undergoing safety and efficacy assessments, the fifth generation of CARs is a derivative of the second generation with an additional IL-2 receptor-chain fragment (IL-687 2R). The IL-2R fragment has a binding site that can activate the JAK-STAT signaling cascade. Once the CAR-T cells identify tumor antigens, the receptor’s antigen-specific activation can simultaneously activate all the downstream signaling pathways, enhancing the persistence and full activation of T cells [187,188]. Solid tumors pose several obstacles to CAR-T-cell activity, despite the effective use of this therapy for hematologic malignancies [189,190,191,192]. These challenges include tumor heterogeneity, an unfavorable tumor microenvironment, limited trafficking and infiltration, and toxicities [193,194]. The scalability of CAR-T therapy is best reflected by its utilization in hematologic malignancies. As of 2024, there are six commercially available CAR-T-cell therapies targeting blood-related cancers [195]. Mohamed Abou-el-Enein et al. provided a comprehensive description of the advancements in CAR-T development, encompassing initial trials, capturing interest from pharmaceutical companies, and addressing challenges in the manufacturing process such as time consumption and safety.

### 5.2. CAR-T Therapy Targets in TNBC

Based on their pattern of expression, tumor-specific antigens (TSAs), tumor-associated antigens (TAAs), and cancer germline antigens (CGAs) are the three categories into which tumor antigens are classified [196]. The most suitable tumor antigens are TSAs, since they are expressed solely on the surface of tumor cells [197]. Although TAAs are more prevalent on tumor cells than on normal tissues (e.g., HER2), targeting tumor cells carries a risk of on-target or off-target side effects considering armed CAR-T cells can also attack normal tissues [198,199]. Since CGAs are primarily expressed in the testis and ovaries, their expression is limited to adult somatic tissues [200]. To date, there are several ongoing clinical trials of CAR-T therapy in TNBC. Amongst CAR-T-cell targets are mesothelin (MSLN), cMet, MUC1, TnMUC1, ROR1, and NKG2D Ligand [201,202]. MSLN is highly expressed in about 67% of TNBC and shows potential as a useful target for CAR-T-cell therapy [203]. Encouraging findings in preclinical research and clinical trials notwithstanding, overcoming barriers limiting therapeutic outcomes and clinical applicability is of fundamental importance. To selectively target tumor cells, CAR-T cells must effectively infiltrate solid tumors like breast cancer. This is mostly dependent on the specific interaction between chemokine receptors on the surface of CAR-T cells and the chemokines presented on tumor cells or the tumor microenvironment. Since different cancer cells produce various chemokines, it is important to determine the specific chemokine(s) that a certain tumor secretes to enable T lymphocytes to recognize it [204]. Regretfully, reports of inconsistencies between chemokine receptors on T cells and chemokines on tumor cells were common [205]. To address this issue, two strategies were used: the former involved designing CAR-T cells with better-matched chemokine receptors [206]. The latter involved using oncolytic viruses that contain chemotactic chemokines to promote CAR-T cells’ infiltration into tumors, yet the viruses may also induce immunogenicity [207]. Other strategies include local administration [208], targeting the fibroblast activation protein [209], or building CARs with enzymes that break down the extracellular matrix of tumor cells [210]. Tumor microenvironment immunosuppression is facilitated by the accumulation of immunosuppressive cells, such as T regulatory cells (Tregs) and myeloid-derived suppressor cells (MDSCs), and inhibitory tumor cytokines, particularly transforming growth factor (TGF), in tumor sites [211]. Through the direct construction of T cells with TGF receptors or the indirect introduction of cytokines (IL-2, IL-15, and IL-12) to neutralize immunosuppressive factors, T-cell persistence and efficacy within tumors were enhanced through cytokine inhibition [212,213]. The host immune system’s elimination of T cells and their inability to react to inhibitory cytokines, however, continue to be restrictions. Additionally, CAR-T-cell and checkpoint inhibitors—specifically, anti-PD1 and anti-PD-L1 blockade—showed superior therapeutic results compared to monotherapy; nevertheless, the addition of checkpoint inhibitors may raise the risk of immunogenicity [214]. tumor heterogeneity, or the variation in antigen expression levels and types on the surface of tumor cells, is another hindrance. However, this challenge has been partially addressed by the effective production of multitarget CAR-T cells, which have demonstrated enhanced anticancer activity in preclinical investigations. Bispecific CAR-T cells directed against both HER2 and MUC1 in breast cancer were effectively generated and demonstrated cytotoxic properties. Additionally, anticancer effects were shown by biCAR and triCAR-T cells [215,216]. The multitarget strategy effectively attracted CAR-T cells to the tumor site and raised the likelihood of eradicating a subset of tumor cells; it also reduced the possibility of on-target or off-tumor adverse effects, hence promoting safety [217]. Lastly, one of the main issues impeding the use of CAR-T-cell therapy are toxicities, such as cytokine release syndrome, hypotension, hypoxia, multiorgan toxicity, and encephalopathy syndrome connected to CAR-T cells [218,219].

### 5.3. CAR-T Cell-Derived Exosomes as a Way to Reduce Toxicity on Healthy Cells

The majority of body cells secrete exosomes, which are nanometric membrane vesicles [220,221]. Exosomes are crucial intercellular messengers that transport a variety of essential compounds, such as proteins, mRNAs, and microRNAs [222]. It has been demonstrated in earlier research that T cell-derived exosomes are capable of delivering effector molecules such as granzymes and perforin that target tumor cells directly [223]. According to recent research, all surface membrane components derived from parental cells—including TCRs, 751 CD3, CD8, and CARs—are expressed in isolated CAR-T-cell exosomes [224]. Yang et al. studied the function of CAR-T-cell exosomes in both in vitro and in vivo, targeting MSLN-positive TNBC. Human peripheral blood T cells were isolated and activated before being transduced with the anti-MSLN CAR or a control retroviral vector. Day 7 post-transduction showed that CAR-transduced T cells had higher amounts of the anti-MSLN CAR than did control transduced T cells (respectively 60% and 2%). Anti-MSLN CAR-T cells were then stimulated with MSLN at day 9, and the exosomes were derived at day 14. The surface of the exosomes contained the anti-MSLN CAR (92%), along with other exosome- and T cell-specific markers such as CD63 and CD3. According to these findings, the exosomes and anti-MSLN CAR-T cells shared the same membrane structure. BT-549 and MDA231-MSLN cell lines were tested in vitro, and results indicated that at doses of 50, 100, and 300 g, anti-MSLN CAR-T-cell exosomes considerably outperformed control exosomes in terms of cell killing efficiency. Results also showed that the effector molecules perforin and granzyme B, which can destroy TNBC cells directly, were carried by the anti-MSLN CAR-T-cell exosomes. Both the BT-549 and MDA231-MSLN xenograft tumor models revealed dose-dependent suppression of tumor growth in the mice treated with anti-MSLN CAR-T-cell exosomes, as evidenced by decreased tumor weight and volume. Even at the highest dose, animals injected with anti-MSLN CAR-T-cell exosomes showed no signs of toxicity. There was no discernible variation in the mice’s body weight across all groups. Furthermore, no apparent pathological alteration was found in the liver, spleen, or heart, indicating exosomes can potentially reduce systemic toxicity connected to CAR-T therapy.

In Table 1, we present the summary of novel targeted therapies in breast cancer treatment.

## 6. Discussion

The potential of novel anticancer therapies for triple-negative breast cancer development is currently the focus of numerous scientific studies (Table 1). In vitro and in vivo experimental research, as well as clinical trials, suggest molecular-based approaches in mitigating the development and progression of TNBC cancer. Furthermore, the presented results indicate that focusing on the hallmarks of cancer cells can exert anti-cancerous effects through different mechanisms. Firstly, by targeting dysregulation in cancer cell metabolism, known as the Warburg effect, using for example glycoconjugates as a trojan horse method to deliver drugs to malignant cells, shell-sheddable micelles and Rg3 liposomes based on ginseng interacting with GLUT-1 as other drug delivery systems. Moreover, the Warburg effect, characterized by increased glycolysis even in the presence of oxygen, contributes to the acidification of the tumor microenvironment due to excessive lactate production. This acidosis, coupled with hypoxia resulting from rapid tumor growth, leads to the activation of various pathways, including those involving hypoxia-inducible factor 1 (HIF-1). Therefore, therapeutic strategies explored to target acidosis and hypoxia in cancer treatment include inhibiting key enzymes like CA9, targeting hypoxia-induced pathways, and exploring novel approaches such as bicarbonate treatment to decrease tumor acidity. Another hallmark of cancer cells is metastasis. It remains a significant challenge in TNBC, driven by factors like tumor heterogeneity and the tumor microenvironment. Immunotherapy, particularly CAR-T-cell therapy, is emerging as a promising approach to target TNBC, leveraging the immunogenicity of the tumor and overcoming immune evasion mechanisms. Ongoing clinical trials are exploring the potential of CAR-T therapy in treating TNBC, building upon its established success in addressing hematologic malignancies. While CAR-T therapy continues to demonstrate a growing impact in oncology treatment, there remains a lack of consensus regarding a potency assay or platform capable of accurately predicting clinical response to this innovative therapy. Consequently, further research is imperative to enhance our understanding and refine the application of CAR-T-cell therapy, underscoring the need for continued investigation in this field. The promising results of the novel therapies presented notwithstanding, they have their limitations. The vast majority of them are in the preclinical phase, with results concerning the outcomes of the treatment on cells and mice, whereas the final goal is to render it patient-specific. Each patient responds differently to the applied therapy; thus, lack of data on human trials is a considerable hindrance. Among other drawbacks are the absence of information regarding the optimal and toxic doses, potential side effects, and pharmacodynamic properties. Innovative biologics remain unattainable to the general public cost-wise, necessitate multiple injections, and raise concerns about the impact of long-term chronic immunosuppression. 

## 7. Conclusions

In conclusion, understanding the complex interplay between metabolism dysregulation in Warburg effect, acidosis, hypoxia, and tumor progression is crucial for developing effective therapeutic strategies, particularly in aggressive cancers like TNBC. Targeting these microenvironmental factors holds promise for improving patient outcomes and overcoming therapeutic resistance.

## Figures and Tables

**Table 1:** Summary of novel targeted therapies in breast cancer treatment.

Cancer Hallmark	Therapeutic Strategy	Model	Results	Reference
Deregulating cellular metabolism	Adriamycin-glycoconjugate targeting GLUT1	In vitro MDA-MB-231 and MCF-7 cells	2DG–SUC–ADM showed greater tumor-targeting ability and antitumor activity than free ADM.	[225]
	Oxaliplatin-glycoconjugate targeting GLUT1	In vitro MCF-7 cells	Fluro-substituted oxaliplatin-glycoconjugate exhibited the highest water-solubility among the three tested complexes. The test compound demonstrated a cytotoxicity level towards MCF7 cell lines that was twice as potent as the established clinical drug oxaliplatin.	[226]
	Methotrexate-glycoconjugate targeting GLUT1	In vitro MCF-7 cells; in vivo 4T1 breast cancer mice	GLU–MTX was approximately 17-fold more preferentially accumulated in cancer cells compared to free MTX. GLU–MTX showed less cytotoxicity to healthy cells than MTX. GLU–MTX resulted in a substantial reduction of 74.4% in the growth of 4T1 allograft tumors on day 18, whereas MTX alone led to a much smaller inhibition of tumor growth, approximately 16.2%.	[227]
	Doxorubicin and 8-hydroxyquinoline glycoconjugate loaded into micelles	In vitro MCF-7 cells	Encapsulating drugs into micelles enhanced metabolic stability, improved tumor therapy selectivity, inhibited cancer cell proliferation more effectively, and induced apoptosis.	[228]
	Rg3 liposomes interacting with GLUT-1	In vitro MCF-7 cells	The findings indicated that Rg3 improved the efficiency of liposome uptake and penetration into MCF-7 spheroids.	[229]
Resisting cell death	Inhibiting NDBTs	In vitro MDA-MB-231 cells	NDBT inhibitor-S0859-reduced spheroid growth in MDA-MB231 cell lines by 48%. S0859 increased apoptosis in the core of MDA-MB-231 spheroids.	[230]
Activating invasion and metastasis	NDBT knockdown	In vitro MDA-MB-231 cells	NDBT knockdown significantly reduced pHi in MDA-MB-231 in normoxia and hypoxia, reducing spheroid growth. NDBT knockdown reduced both invasion and migration in normoxia and hypoxia.	[231]
	LOXL2 inhibition	In vitro MDA-MB-231 cells; in vivo orthotopic MDA-MB-231 human breast cancers in mice	Both the dual inhibitor PXS-S1A and the LOXL2-specific inhibitor PXS-S2A suppressed cellular proliferation in a dose-dependent manner over an 8-day period in vitro. PXS-S1A exhibited an approximately 75% reduction in primary tumor volume, while PXS-S2B demonstrated a decrease of around 55% in tumor volume in vivo.	[232]
	Lipid nanovesicles targeting LOX	In vitro MDA-MB-231 cells; in vivo orthotopic MDA-MB-231 human breast cancers in mice	The delivery of EPI was significantly enhanced at both time intervals (6 and 48 h) when it was encapsulated within Lipo-EPI-LOX particles compared to free EPI and Lipo-EPI in vitro. Lipo-EPI-LOX displayed a substantial reduction in tumor growth compared to Lipo-EPI and free EPI in MDA-MB-231 murine xenografts over a 33-day period. Results demonstrated that Lipo-EPI-LOX treatment was the best tolerated therapy.	[233]
Avoiding immune destruction	“BiCyclA”	In vivo 67NR, 4T1-Luc, E0771 breast cancer mice	While BiCyclA effectively cured most mice with 4T1-Luc, 67NR, and E0771 tumors, only mice bearing E0771 tumors fully resisted tumor rechallenge.	[234]
	CAR-T cell-derived exosomes	In vitro MDA-MB-231 cells; in vivo orthotopic MDA-MB-231 human breast cancers in mice	The anti-MSLN CAR-T-cell exosomes demonstrated notably greater efficacy in killing cells in vitro compared to control exosomes when targeting MDA231-MSLN cell lines. ELISA results suggested that the anti-MSLN CAR-T-cell exosomes contained effector molecules, such as perforin and granzyme B, capable of directly targeting and killing TNBC cells. The results indicated that anti-MSLN CAR-T-cell exosome treatment effectively inhibited tumor growth in MDA231-MSLN xenograft models without causing toxicity at any dosage.	[235]
